# Breath Biopsy and Discovery of Exclusive Volatile Organic Compounds for Diagnosis of Infectious Diseases

**DOI:** 10.3389/fcimb.2020.564194

**Published:** 2021-01-07

**Authors:** José E. Belizário, Joel Faintuch, Miguel Garay Malpartida

**Affiliations:** ^1^ Department of Pharmacology, Institute of Biomedical Sciences, University of São Paulo, São Paulo, Brazil; ^2^ Department of Gastroenterology of Medical School, University of Sao Paulo, São Paulo, Brazil; ^3^ School of Arts, Sciences and Humanities (EACH), University of Sao Paulo, São Paulo, Brazil

**Keywords:** breath biopsy, infectious disease, microbiomes, metabolomics, biomarkers

## Abstract

Exhaled breath contains thousand metabolites and volatile organic compounds (VOCs) that originated from both respiratory tract and internal organ systems and their microbiomes. Commensal and pathogenic bacteria and virus of microbiomes are capable of producing VOCs of different chemical classes, and some of them may serve as biomarkers for installation and progression of various common human diseases. Here we describe qualitative and quantitative methods for measuring VOC fingerprints generated by cellular and microbial metabolic and pathologic pathways. We describe different chemical classes of VOCs and their role in the host cell-microbial interactions and their impact on infection disease pathology. We also update on recent progress on VOC signatures emitted by isolated bacterial species and microbiomes, and VOCs identified in exhaled breath of patients with respiratory tract and gastrointestinal diseases, and inflammatory syndromes, including the acute respiratory distress syndrome and sepsis. The VOC curated databases and instrumentations have been developed through statistically robust breathomic research in large patient populations. Scientists have now the opportunity to find potential biomarkers for both triage and diagnosis of particular human disease.

## Introduction

Trillions of microbes mutually coexist in different sites of human body, especially in the gut, to fulfil our cells’ nutrient demands (O’Connor, 2013; Rowland et al., 2018). The healthy to diseased transition frequently results from the disruption of diversity of microbe species living in symbiotic niches. Alterations (dysbiosis) of the microbial community equilibrium can result in the outgrowth of pathogenic species and suppression of commensal species, a signal to our body to initiate an inflammatory attack to microbes and host cells. Microbial dysbiosis has been a postulated pathway to many diseases, including obesity, inflammatory bowel disease (IBD), type 1 diabetes (T1D) and type 2 diabetes(T2D), inflammatory airway diseases, rheumatoid arthritis (RA), allergy, autism, and cancer (Clemente et al., 2012; Belizario and Napolitano, 2015). There have been many attempts to define whether an individual bacteria specie or an enriched or depleted genera contributes to dysbiosis in healthy and diseased states. Studies on enriched or depleted operational taxonomic units (OTUs) identified the genera Bacteroides, Prevotella, and Ruminococcus as the most common dysbiotic taxa in common chronic diseases (Wilkins et al., 2019). For instance, hierarchal clustering reveals that *Bacteroides* genus is associated with urinary stone disease and *Blautia* genus with diabetes (Wilkins et al., 2019). The human microbiomes harbor a rich and diverse array of biosynthetic and biochemical pathways. Thus, through diverse enzyme-catalyzed processes, bacteria can produce a larger variety of bioactive molecules as compared to metabolic enzymes operating in hundreds of types of cells that make up our organs and tissues. The culture-based method, contrary to non-culture-based methods, can only identify a small group of microbial species. Thus, understanding of the entire microbial community and their network dynamic throughout the enzymes-mediated metabolism associated with metabolic phenotypes in complex niches is limited.

**Figure 1:**
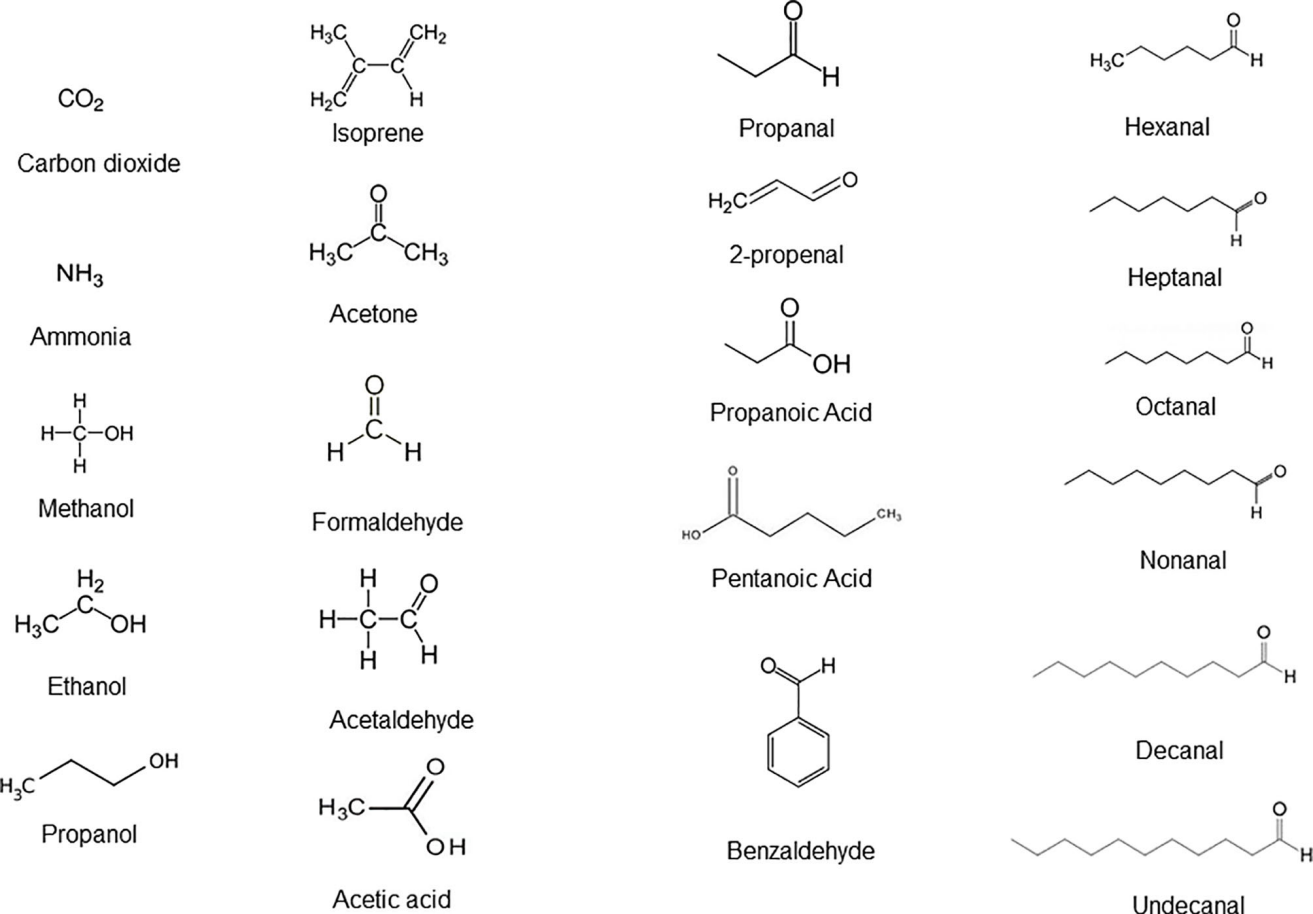
Different classes of endogenous molecules and VOCs detected in a simple whole breath sample that are biochemically produced upon carbohydrates and fatty acid metabolism in cells of the organ systems and bacterial species of the microbiomes under healthy state. Adapted from Doran et al., 2018.

In the last decade, thousands of soluble and volatile small molecules representing functional activity of both microbiome and host cell metabolomes were discovered and catalogued. VOCs are in general the end products of carbohydrate metabolism and lipid metabolism as well as oxidative stress and cytochrome p450 liver enzymes in the human cells, as well as aerobic and anaerobic fermentation processes of bacteria living in the gut microbiomes. **Figure 1** presents a list of 21 chemically relevant endogenous metabolites and VOCs which are commonly detected in whole expiratory human breath and represent the reference standard of VOC analysis. Under physiological conditions, VOCs such as acetate, propionate, cis-2-methylcrotonate, 2-methylbutyrate and 2-methylvalerate, short chain fatty acids (SCFAs), alcohols, propanols, hydrocarbons, aldehydes, ketone terpenes, acids, nitrogen and sulfur-containing compounds are emitted in the exhaled air, feces, and body fluids (Audrain et al., 2015; Rees et al., 2018). Nitric oxide (NO), carbon dioxide (CO_2_), carbon monoxide (CO), hydrogen cyanide (HCN), and hydrogen sulfide (H_2_S) are inorganic and endogenous gaseous transmitters involved in the regulation of many biological processes (Shatalin et al., 2011). H_2_S is oxidized into thiosulfate and then into tetrathionate by the colonic epithelium of the colon (Shatalin et al., 2011). Tetrathionate is a terminal electron acceptor during anaerobic respiration (Ribet and Cossart, 2015). It serves as a substrate for methane synthesis, one of the most abundant gas in environment. Gram-positive and gram-negative bacteria produce indole in large quantities. This metabolite enters into tryptophan biosynthesis, which is an amino acid that serves as an intercellular and extracellular signal in microbial communication. Indole is essential for biofilm formation (Lee et al., 2007). A large set of biologically active small molecules and peptides can modulate the transcription of genes in response to local changes in cell number and density, a phenomenon known as quorum sensing (QS) (Rutherford and Bassler, 2012; Belizario et al., 2020).

Over 2000 VOCs emitted by microbes and human cells have been identified and catalogued according to key classes and chemical structures (Broza et al., 2014; de Lacy Costello et al., 2014; Vinaixa et al., 2016; Vizcaino et al., 2016; Lemfack et al., 2017). There are few VOCs with relatively lower concentrations (below 1 ppb) in exhaled breath. Principal component analysis (PCA), partial least-squares to latent structures (PLS), and orthogonal PLS (OPLS) are some examples of bioinformatics tools used for analyses, validation, and robust modeling of the epidemiological and chemical data sets for discovery of unique biomarkers and signatures. The heat map is another way for clustering data and to find potential links between chemical structure and the biological activities of VOCs. Standardized repositories such as mVOC (http://bioinformatics.charite.de/mvoc) and Metabolite Ecology DB (http://kanaya.naist.jp/MetaboliteEcology/top.jsp) have allowed both quantitative and qualitative analyses of VOCs in their biochemical pathways and biological roles in the healthy and diseased (de Lacy Costello et al., 2014; Abdullah et al., 2015). Future studies are needed for deciphering mVOC-mediated microbe–microbe interactions. We do not know if endogenous or exogenous VOCs bind to specific receptors and are internalized and transduced by receptor-mediated processes, or if they interact with the cell membrane lipids to initiate signal transduction cascades or if they are simply taken up by cells and metabolized.

Breath biopsy is a term that refers to VOC sampling from exhaled air. Various types of instruments and methods have been used to analyze chemically and molecularly distinct VOCs. The thermal desorber associated with gas chromatography and quadrupole mass spectrometry (TD-GC-MS), proton transfer reaction mass spectrometry (PTR-MS), and electronic nose sensors (eNose) are examples of current technologies for detection and quantification of physiologically and pathologically relevant VOCs (Dragonieri et al., 2017). A small number of exclusive VOCs were found that most strongly mirror gut microbial diversity and potential infection in the cohort healthy and diseased patients (de Lacy Costello et al., 2014; Palma et al., 2018). The profiles of VOCs can distinguish infections from each other and from viruses and fungi and may be useful in monitoring microbial infection and monitoring clinical response to drug treatment. However, many clinical and methodology challenges have been encountered to compare and verify the uniqueness of the exhaled VOC profiles for a specific disease (Stavropoulos et al., 2020). VOC biopsy does not appear to be ready yet for implementation as a medical diagnostic tool (Stavropoulos et al., 2020). Here we first give an overview on the breath sample biopsy and gas chromatography and mass spectrometry methods for chemical detection of VOCs. Next, we describe protocols and approaches currently available, or under development, for discovery and monitoring of the diagnosis of common diseases of the respiratory tract, gastrointestinal disorders, and sepsis. The aim of this review is to anticipate the current advances in VOC curated chemical metadata that will assist basic and clinical laboratories to quickly and precisely detect human diseases, thus providing physicians with critical information for timely and appropriately management and treatment of patients.

## Breath Biopsy Instrumentation

Exhaled breath or expiratory breath consists of a mixture of nitrogen (78%), oxygen (13%), carbon dioxide (5%), water vapor (4%), inert gases, and thousands volatile compounds with low molecular weight (less than 500 Da) (de Lacy Costello et al., 2014). Systemic VOCs travel efficiently from the blood into the alveolar air and continue through the respiratory tract—this includes lungs, pharynx, larynx, nose, oral cavity, sinuses—and finally to move out to the external environment. Breath biopsy is a method of collecting gaseous molecules from our own endogenous metabolisms. Breathomics refers to repertory of gases and VOCs derived from specific cellular and tissue metabolism of the host cells and local microbiomes (Amann et al., 2014; de Lacy Costello et al., 2014).

A large number of VOCs, proteins, and peptides identified in water condensates, respiratory droplets, and exhaled breath aerosols have been indicated as measurable biological markers for the diagnosis of oxidative stress, inflammation, carcinogens, and microbial infection (Boots et al., 2012; Amann et al., 2014; de Lacy Costello et al., 2014; Ahmed et al., 2017; Timm et al., 2018). The ammonia breath tests using ^13^C-urea is a most reliable technique for clinically detecting *Helicobacter pylori* infection. The exhaled nitric oxide (NO) level test can help diagnose asthma, whereas the acetone levels can help diagnose diabetes mellitus and ketonemia, e.g., increase in ketone bodies. Measurement of isoprene and ammonia levels is used to access renal disorders (Ulanowska et al., 2011). VOCs are also emitted from biological fluids such as blood, saliva, skin, milk, and feces. VOC emission from these resources is concentrated in headspace, inert polymer bags, or directly onto thermal adsorbent tubes such as Tenax TA, Carbotrap, and other sorbent materials (Amann et al., 2014; Herbig and Beauchamp, 2014). Owlstone Medical (Cambridge, UK) has developed devices called ReCIVA (Respiration collector for *in vitro* analysis) and CASPER (Clean air supply pump) and standardized procedures to capture, store, and analyze breath biopsy samples. Breath biopsy is non-invasive and requires no patient efforts. The volumes of exhaled air are collected according to the appropriate CO_2_ levels using active pumps that guide gases onto two or four special adsorbent tubes placed into the ReCIVA device. Various groups have set up the platforms and devices for discovery and validation of VOCs for diagnostic or controlling therapeutic responses in cohorts of human patients. A standardized method for optimization of breath biopsy sampling using these devices and critical parameters for data analysis is presented in detail in a recent paper (Doran et al., 2018). **Table 1** briefly describes some devices and instrumentations for VOC sampling and off-line and on-line chemical analyses and compares some advantages and limitations of their clinical application.

**Table 1:** Breath biopsy tools and instrumentations for analysis of volatile organic compounds.

Sampling methods	Extraction capability	Advantages/Limitations
Solid-phase micro extraction resins (SPME)	Low to medium	Performance depending on fiber type and affinity to targeted molecules
Thermal adsorbent tubes	High	Robust and high performance for VOCs extraction
Gas sampling bags	Low	Performance depending on the material, high permeability to VOCs
Respiration collector for *in vitro* analysis (ReCIVA)	High	Clinical use, high performance, easy sampling, low environment gas contamination
**Analytical methods**	**Sensibility**	**Advantages/Limitations**
Thermal desorption, gas chromatography and triple and quadrupole mass spectrometry (TD-CG-MS)	ppb	Relatively easy to use, standardized method for clinical VOC measurement, low cost
Two-dimensional gas chromatography, mass spectrometry, time of flight (GCxGC-MS-TOF)	ppb	Relatively easy to use, standardized method for clinical VOC measurement, low cost
Selected ion flow tube (SIFT)	ppb to ppt	Direct detection, relies on reaction with reagent ion, high cost, needs a specialist
Proton transfer reaction with mass spectrometry (PTR-MS, PTR-TOF-MS)	ppb to ppt	Relies on reaction with reagent ion, high cost, needs a specialist
Ionic molecule reaction with mass spectrometry (IMR-MS)	ppm to ppb	Allows detection of mixtures of small molecules with low fragmentation and chemical selectivity
Ion mobility spectrometry (IMS)/field asymmetric ion mobility spectrometry (FAIMS)	ppm to ppb	Portable, easy to use and affordable, ideal for use in point of care applications
Electronic nose sensors (eNose)	ppm	Relies on VOC reaction with selective sensors and chemicals, portable, easy to use

ppm, parts per million; ppb, parts per billion; ppt, parts per trillion.

The most common chemical detection methods for VOC analysis is the gas chromatography (GC) associated with mass spectrometry (MS). Through various steps, these methods separate and identify the individual constituents of a gaseous sample, but to be quantitative, the technique requires calibration with commercially available synthetized pure form of the target compound. Gas pre-concentration require devices such as thermal desorption (TD) system, solid-phase microextraction (HS-SPME), or needle trap devices, which can enhance collection and detection of targeted VOCs. TD carries out a controlled heating process to release the captured VOCs from adsorption tubes. TD-GC-MS method of thermally stable volatile compounds is appropriate for identification of alcohols, aldehydes, esters, terpenes, thiols, or aromatic compounds. A mass spectrometer is composed of a source, an analyzer, and a detector. The source promotes the ionization of molecules, an analyzer separates all metabolites and identify each metabolite by their mass-to-charge (m/z) ratio, and a detector registers the relative number of counts per hit. This process is particularly suited for identification of unknown molecules.

Different types of mass spectrometry analyzers are commercially available. The time of flight (TOF) is the most used in the mass spectrometers because of its mass accuracy that vary from several part per million (ppm) of error. The quadrupole time of flight (QTOF) technology enables the ion separation and subsequent collision-induced dissociation and identification of fragmented ions. The triple quadrupole (QqQ) consists of two quadrupole mass analyzers in series that allow target quantification by multiple reaction monitoring (MRM) mode. In GC-MS method, the identification of the metabolic chemical features is definite based on the retention times and spectra from empirical data to internal reference library or by comparing their accurate masses in one chemical database (Gowda et al., 2014). Many public database servers such as online chemical databases HMDB (https://hmdb.ca/), METLIN (https://metlin.scripps.edu/), and NIST (http://chemdata.nist.gov) are available for retrieval and analysis of data online.

Proton transfer reaction (PTR)-MS and selected ion flow tube (SIFT)-MS, mass spectrometry with ionic molecule reaction (IMR-MS), ion mobility spectrometry (IMS), and field asymmetric ion mobility spectrometry (FAIMS) are novel technologies which allow direct injection of samples for detection and quantification of VOCs. The ion mobility spectrometry is a chemical method in which an ionized sample interacts with one carrier buffer—an inert gas—in the presence of weak electric field to produce a separation and identification of the analytes according to their size, shape, and charge. Ion mobility spectrometry can work in combination with other mass analyzers. The Lonestar is a field asymmetric ion mobility spectrometry developed by Owlstone Medical, UK, used for profiling and identification of VOCs collected from breath biopsy (Arasaradnam et al., 2013). All these chemical analytical approaches are adequate for monitoring highly volatile species in breath in real-time, providing immediate chemical data per billion levels of sensitivity without the requirement for pre-concentration procedures. One of the recent advance in VOC analyses is the application of two-dimensional (2-D) GC (GC-GC) and 2-D MS (MS-MS) in combination with fast MS analyzer, such as time of flight (TOF) (Phillips et al., 2013). This method enhances the resolution and quantification of VOC biomarker candidates (Phillips et al., 2013).

**Figure 2:**
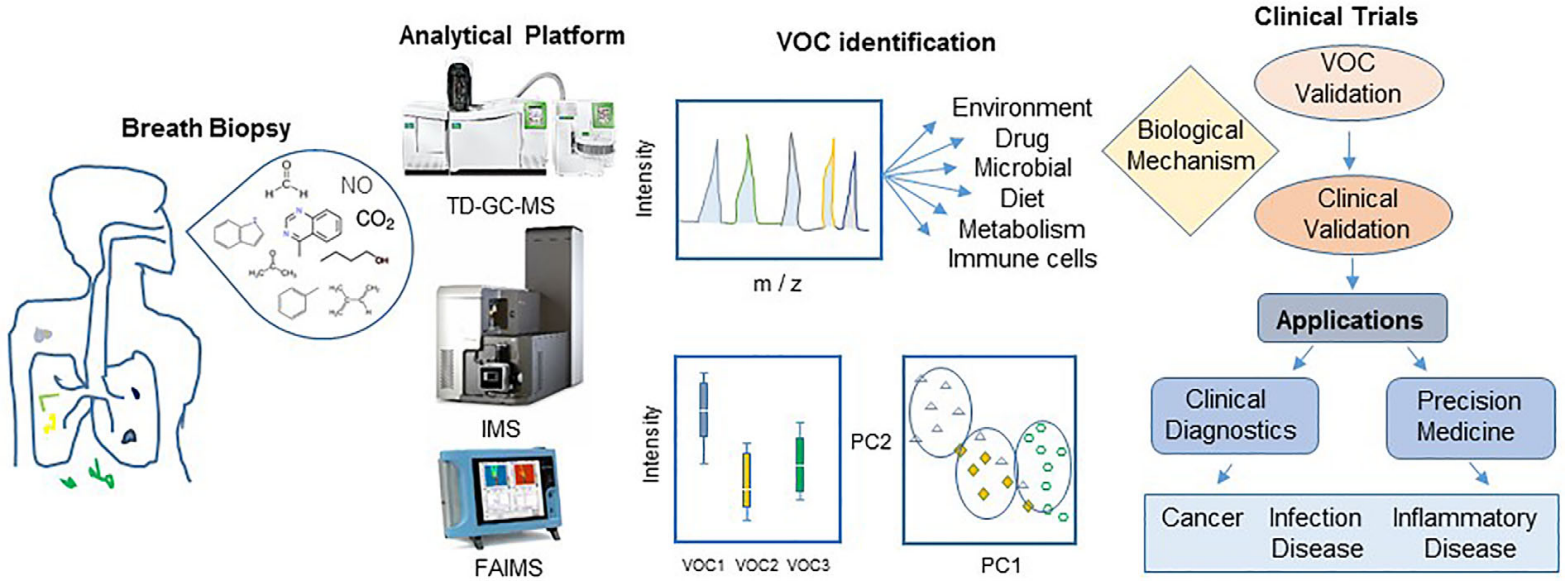
Workflow for breath biopsy and VOC discovery for disease diagnosis. An exploratory study starts with careful design of protocols for breath biopsy and platform analysis of wide variety of compounds resulting in a panel of potential biomarkers. Unsupervised and supervised approaches such as principal components analysis (PC) should be followed by validation experiments to generate clinically reliable biomarkers for their application in medical practice. Abbreviation: TD-GC-MS, thermal desorption-gas-chromatography mass spectrometry; IMS, ion mobility mass spectrometry (IMS); FAIMS, field asymmetric ion mobility spectrometry.

Diverse brands of portable chemical, gas sensors and electronic noses (eNose), such as Cyranose C320, Tor Vergata eNose, CSA, based in metal-oxide colorimetric sensor arrays and electron chemical sensors have been developed and are commercially available (Wilson, 2015). Apparatus and devices for collecting, concentrating, separating, and identifying breath proteins, metabolites, and VOCs as well as for batch variation and correction, inter-instrument analytical differences have been the subject of extensive study and reviews (Wilson, 2015; Ahmed et al., 2018; Doran et al., 2018). Several standard multivariate statistical and bioinformatics methods, including the unsupervised and supervised approaches such as principal components analysis (PCA), hierarchical clustering analysis (HCA), projection to latent structures (PLS), pattern recognition analysis, and fuzzy logic analysis have been recommended for profiling targeted and untargeted analyses of potential VOC biomarkers in exhaled air from experimental and prospective longitudinal clinical studies (Ahmed et al., 2018; Doran et al., 2018). A typical workflow for exhaled breath analysis and VOC biomarker validation for medical diagnosis is shown in **Figure 2**.

## VOC Signatures Emmited by Clinically Relevant Bacterial Species

**Figure 3:**
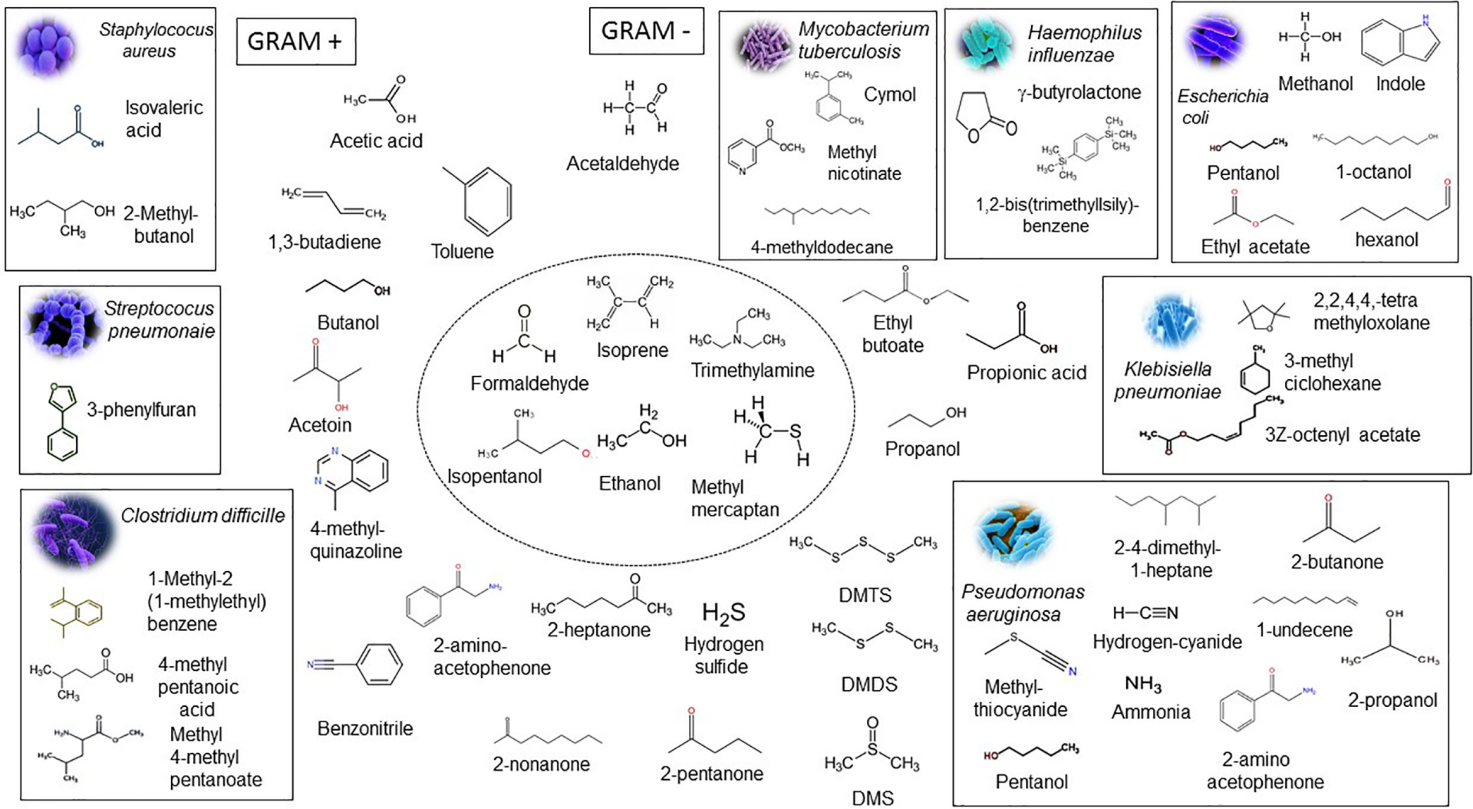
Microbial VOC signatures. The rectangle contains chemical structures and names of the most prominent VOCs that characterize the presence of gram-positive bacterial strains *Staphylococcus aureus*, *Streptococcus pneumoniae*, and *Clostridium difficile* and gram-negative bacterial strains *Escherichia coli, Klebisiella pneumoniae, Pseudomonas aeruginosa*, *Haemophilus influenzae*, and *Mycobacterium tuberculosis* identified in a large cohort of patients in the course of the infection. The central circle shows representative VOCs produced by all bacteria. Isopentanol, formaldehyde, methyl mercaptan, and trimethylamine are produced only by bacteria and not by the eukaryotic cells. VOCs outside of rectangles are emitted by the host cells and bacteria and considered as sub-products or intermediates of the metabolic pathways. Acetaldehyde, ethanol, and isoprene are found in large amounts in human exhaled breath. The inorganic compounds and sulfur-containing compounds are associated with an inflammatory process and include ammonia, nitric oxide, hydrogen cyanide, hydrogen sulfide, dimethyl sulfide, dimethyl disulfide, and dimethyl trisulfide. Adapted from Bos L. D. J. et al., 2013, and Palma et al., 2018.

Bacterial species are identified in the clinical laboratory by morphological traits and biochemical and cultural tests. More recently, bacterial species identification has been done through a specific gene, rRNA fingerprinting, and whole DNA sequence (Belizario and Napolitano, 2015). The chemical release of metabolites and VOCs represent a good alternative for the analysis of bacterial species from the clinical specimen and application in different areas. Putative VOC signatures for many pathogens were identified in systematic review and meta-analysis of the results of published studies in the period of 1977 up to 2016 and mentioned in the articles (Bos L. D. J. et al., 2013; Sethi et al., 2013; Ahmed et al., 2017; Ratiu et al., 2017; Palma et al., 2018). VOC microbial signatures have been utilized in predicting, diagnosing, and monitoring infections, dysbiosis, and antimicrobial treatment and resistance (Bos L. D. J. et al., 2013; Sethi et al., 2013; Ahmed et al., 2017; Ratiu et al., 2017; Palma et al., 2018). Palma and colleagues developed a machine learning algorithm based on a database with 792 VOCs that specifically predict with high accuracy and precision the presence of bacteria, protozoa. and fungi during their growth in various *in vitro* and *in vivo* conditions (Palma et al., 2018). A set of VOC microbial signatures were identified to be associated with bacterial and fungal diseases as compared to either patients and healthy control studies in comparison to *in vitro* microbial culture headspace experiments. **Figure 3** displays names, chemical structures, and microbial pathogens of relevant gram-negative and gram-positive bacterial strains, in which a putative VOC signature have been assigned. A common pattern of VOCs contained isopentanol, formaldehyde, methyl mercaptan, and trimethylamine and was emitted by all species of bacteria (central circle of **Figure 3**). A set of VOCs was identified as unique or most representative for a bacterial species (gram positive or negative). In particular a VOC signature assigned to *Staphylococcus aureus* is associated with the following VOCs: isovaleric acid and 2-methyl-methylbutanol; for *Pseudomonas aeruginosa*: 1-undecane, 2, 4-dimethyl-1-heptane, 2-butanone, 2-propanol, ammonia, 2-acetophenone, hydrogen cyanide, and methylthiocyanide; for *Escherichia coli*: methanol, pentanol, ethyl acetate, indole, 1-octanol and hexanol; 2,2,4,4,tetramethyloxolane, 3Z-octenyl acetate and 3-methylcyclohexene; for *Klebsiella pneumoniae*: methyl 4-methylpentanoate, 4-methylpentanoic acid and 1-methyl-2-(1-methylethyl)-benzene; for *Clostridium difficile*: cymol, 4-methyldodecane, methyl nicotinate and 4-methyldodecane for *Mycobacterium tuberculosis*; 1,2-bis(trimethyllsily)-benzene; and for *Haemophilus influenzae*: γ-butyrolactone (Filipiak et al., 2012; Bos et al., 2013; Ahmed et al., 2017; Palma et al., 2018; Belizario et al., 2020). A VOC microbial signature may vary in the patient cohorts in comparison to signature identified in *in vitro* microbial culture headspace experiments. For example, *P. aeruginosa* produces longer chain VOCs, such as 2-undecanone and 2-undecanol in higher amounts at 37°C than 30°C (Timm et al., 2018). VOC signature of eight microbial strains representing genera in the human skin microbiome were identified after 7 days of incubation in multiple media types (Timm et al., 2018). The majority of the results are tightly connected to biosynthetic pathways of bacterial species and VOC emitted by infected patients, reflecting the unique metabolic state of an organism in specific environments. Nonetheless, many bacterial VOC signatures have not been validated sufficiently in large-scale clinical studies for application in diagnostic test (Sethi et al., 2013). Therefore, VOC microbial signatures for each bacterial genera and species need to be well established using a standardized instrumentation and normalization methods to ensure potential clinical translation (Ahmed et al., 2018; Doran et al., 2018).

## VOC Signatures Emitted by Respiratory Tract Microbiome and Diseases

Diverse and dynamic bacterial communities live in the upper (nasal, mouth, trachea, and upper bronchus) and lower (lungs, bronchi, bronchioles, and alveoli) respiratory tracts (Huffnagle et al., 2017, Moffatt and Cookson, 2017). Respiratory tract microbiome is colonized mainly by Bacteroidetes and Firmicutes phylum and bacterial species of genera *Streptococcus*, *Prevotella*, *Veillonella*, *Fusobacterium*, and *Haemophilus* (Huffnagle et al., 2017; Moffatt and Cookson, 2017). The lung microbiome has important roles in the major respiratory diseases and inflammatory conditions including chronic obstructive pulmonary disease (COPD), asthma, and acute respiratory distress syndrome (ARDS) (Barnes, 2017; Lamarche et al., 2018; Mendez et al., 2019). COPD and asthma are respiratory diseases with multiple phenotypes and endotypes usually produced by genetic background, exposure to respiratory viruses, bacteria co-infection, and inhaled noxious environmental pollution (Barnes, 2017; Lamarche et al., 2018; Mendez et al., 2019). ARDS is a common disease in critically ill patients. ARDS develops from an acute-onset of tissue hypoxia that is followed by lung infiltration, diffuse alveolar damage, chaotic inflammation, activated coagulation, and lung fibrosis. ARDS patients have very poor outcomes.

Lung infections share common clinical features with community-acquired pneumonia (CAP). CAP is caused by various bacteria strains including *Streptococcus pneumonia*, *M. tuberculosis, Legionella pneumophila, S. aureus, H. influenzae, Coxiella burnetii*, and other species. Some of these bacterial species are present in the airways of healthy subjects as well as asthma and COPD patient cohorts (Brinkman et al., 2015; Nizio et al., 2016; van Vliet et al., 2017; Ahmed et al., 2018; Kuruvilla et al., 2019). The symptoms of viral and bacterial pneumonia overlap. Moreover, it has been difficult to distinguish the clinical symptoms of viral infection caused by influenza A virus, respiratory syncytial virus, picornavirus, parainfluenza viruses, hantavirus, and coronaviruses (Molyneaux et al., 2013). The heterogeneity of these diseases require new methods for discovery and validation of novel microbial metabolites that are generated during airway microbiota shifts and their relationship with common alveolar and plasma biomarkers and disease phenotypes and endotypes (Walter et al., 2014; Huffnagle et al., 2017; Lamarche et al., 2018; Kuruvilla et al., 2019).

After initial infection, viruses and pathogenic bacteria induce inflammation causing the increase in the mucus production. Airway inflammation increases the production of reactive oxygen species (ROS) and reactive nitrogen species (RNS) in immune cells. These reactive compounds are responsible for damaging of cell membranes and tissue destruction and degeneration. RNS are produced *via* inducible nitric oxide synthase (iNOS). Numerous inflammatory cytokines and protein biomarkers of the coagulation and fibrinolytic cascades and endothelial and epithelial cell injury have been associated with both the development and progression of lung diseases. Many studies have analyzed the relationships between the levels of plasma biomarkers linked to lung tissue injury and their association with mild pulmonary tissue damage and fatal ARDS (Walter et al., 2014; Lamarche et al., 2018). The fractional exhaled nitric oxide (FE_NO_) that originated from NO production by iNOS is a nonspecific biomarker associated with cytokines IL-5 and IL-13 expressed by activation in the epithelial cells. FE_NO_ and CO are potential biomarkers for the diagnosis of airway inflammation and oxidative stress in the lung. In addition, high FE_NO_ values occur during or after respiratory tract infections. Levels of FE_NO_ are significantly higher in patients with chronic rhinosinusitis and allergic rhinitis as compared to patients with no allergic rhinitis (Rolla et al., 2007). A study evaluated the concentration of 16 VOCs belonging to the chemical classes acetone, aldehydes, fatty acids, and phenols in the exhaled breath of healthy volunteers (Doran et al., 2018). These VOCs were detected with high reproducibility in lower airways expiratory breath and whole expiratory breath collected using a standardized breath biopsy device (Doran et al., 2018). The simultaneous analysis of a wide spectrum of breath VOCs offers new perspectives for clinical studies aiming at validating biomarkers for diagnosing lung diseases. Moreover, monitoring of VOC variation in exhaled breath may be useful for the assessment of treatment efficacy of pathological pulmonary processes. Surfactants are tensoactive substances expressed by alveolar type 2 epithelial cells that exert important role in the stability of the alveoli structure and function as well as mechanical strength for gas exchange during breathing (Serrano and Pérez-Gil, 2006). Deficiency, dysfunction, or inactivation of surfactant active ingredients has been shown to trigger or aggravate various modalities of lung infection or injury, including cystic fibrosis associated lung infections, bronchiolitis, and acute respiratory distress syndrome (Al-Saiedy et al., 2018). Surfactant is composed of both saturated and unsaturated lipids (mainly in the form of phospholipids) and proteins. Saturated and particularly polyunsaturated fatty acids and their metabolites circulate in plasma and disseminate in bronchi-alveolar fluid (Al-Saiedy et al., 2018). A variety of VOCs derived from peroxidation of omega-6 (ω-6) fatty acids (linoleic and arachidonic acid), such as n-pentane, epoxides, ketones, acids, and aldehydes were identified as putative biomarkers of lung damage, asthma, cystic fibrosis (CF), and COPD (Phillips et al., 2003; Ibrahim et al., 2011; Bos et al., 2013; Smolinska et al., 2014; Neerincx et al., 2017; Ahmed et al., 2018). VOCs of nonpulmonary origin (blood-borne VOCs) from the breath and from the extracorporeal circulation of critically ill patients can be measured (van Oort et al., 2017; Leopold et al., 2019). Electronic noses, needle trap micro extraction (NTME) devices, and gas sampling pumps can be connected directly to ventilators to collect VOCs from mechanically-ventilated patients undergoing ventilator associated pneumonia (VAP) (Hüppe et al., 2017; Leopold et al., 2019). Octane, acetaldehyde, and 2,3-methylheptane were identified as volatile biomarkers for ARDS (Bos et al., 2014). Elevated pentane concentrations indicate oxidative stress in VAP patients, whereas reduced aldehyde concentrations indicate chemical quenching through reactive oxygen species and peroxynitrite (ONOO) produced in the alveoli of these patients (Bos et al., 2014). Several VOCs are produced by eosinophils and neutrophils. Acetaldehyde is a product of bacterial metabolism, and also leukocytes and neutrophils. Airway inflammation caused by infiltration of eosinophils increases the risk of severe exacerbations, and the inflammatory biomarkers are used for monitoring the responsiveness of asthma patients to inhaled corticosteroids (Brinkman et al., 2015). Exhaled hydrocarbons, for example hexanal, are predictive biomarkers of asthma exacerbations in childhood. Nevertheless, the inventory of biomarkers for asthma and COPD is not firmly established and understood (Neerincx et al., 2017). Patients under higher tidal volume ventilation are at high risk to develop additional ventilator-induced lung injury. In this context, exhaled air may contain contaminants from intensive care ventilators, compressed air and oxygen released from the central gas supply and cylinders, and ambient air of intensive care units. Therefore, more studies to investigate the precise correlations among variables and specific VOCs, plasma biomarkers, and outcomes of lung disease patients at bedside are obligatory (Hüppe et al., 2017; Brinkman et al., 2019).

Cystic fibrosis (CF) is a genetic disease caused by a mutation in the CFTR gene (cystic fibrosis transmembrane conductance regulator). CF patients suffer frequently from pulmonary infections that include the pathogen species *S. aureus*, *H. influenzae*, *Burkholderia cepacia*, *P. aeruginosa*, and other species (Nizio et al., 2016; Mendez et al., 2019). The level of ethane is significantly higher in CF patients, and the levels correlate directly with airway obstruction and increased released of carbon monoxide. Studies on cohort of CF patients revealed that hydrogen cyanide, methyl thiocyanate, and 2-aminoacetophenone are potential breath biomarkers for diagnosis of *P. aeruginosa* infection (Gilchrist et al., 2015).

Tuberculosis (TB) is a chronic disease whose main cause is the infection by bacillus *M. tuberculosis* (MTB). The mycobacteria infection can spread into lungs as well as kidneys, spine cord, and brain. Higher levels of o-xylene and isopropyl acetate and decreased levels of 3-pentanol, dimethylstyrene, and cymol were found in the urine of TB patients compared to healthy controls (Lim et al., 2016; Palma et al., 2018). Other known pathogenic mycobacteria cause TB and lung diseases. More than 130 VOCs were identified in the culture headspace of 13 mycobacterial species during the growth under standardized conditions (Küntzel et al., 2018). One study has identified a core-signature of VOCs emitted by 17 mycobacterial species under optimized bacterial culture *in vitro*, which can be used to established a diagnostic protocol (Küntzel et al., 2018). The dataset comprised of 17 different VOCs revealed that the levels of 2-methylpropanol, 2-methyl-1-butanol, pentane, heptane, octane, 2,3-butadione, 3-pentanone, and 3-octanone were higher in growing cultures whereas the levels of acetaldehyde, propanal, 3-methylbutanal, 2-methylbutanal, pentanal, hexanal, heptanal, benzaldehyde, and 2-methylpropanal decreased below control levels along the incubation period of two or four weeks (Küntzel et al., 2018). This study could not identify a good biomarker for the presence of MTB. Further studies are required for clinical diagnostic application of VOC signatures of TB in clinical setting.

Elucidating VOC signatures emitted after infection by influenza A virus, metapneumovirus, rhinoviruses, and coronavirus would allow timely diagnosis and intervention for respiratory infection and ARDS (Abd El Qader et al., 2015; Rosas-Salazar et al., 2016). Distinct VOC signatures were identified in the headspace of the cultured HEp-2 cells during co-infection with the three bacterial and five viral strains (Abd El Qader et al., 2015; Nizio et al., 2016). Heptane and methylcyclohexane were associated with bacterial infection activity, whereas 1-hexanol and 1-heptadecene were associated with virus infection (Abd El Qader et al., 2015). There is increasing interest to test if these microbial VOC signatures may serve to predict etiology and severe exacerbation and guide antimicrobial or antiretroviral therapy of acute and chronic respiratory infections in children and adults. A study evaluated the changes of VOC breath signature after inoculation of an influenza subtype A (H1N1) vaccine in the groups of heathy volunteers (Phillips et al., 2010). It was shown that 2,8-dimethyl-undecane had a positive correlation with vaccine response over time (Phillips et al., 2010). FE_NO_ and isoprene, a biomarker for influenza virus, increase daily after administering the H1N1 2009 monovalent live intranasal vaccine (Mashir et al., 2011). A recent study identified ethanal, octanal, acetone, butanone, and methanol as VOCs that discriminated SARS-CoV-2/COVID-19 patients and can be predictive for disease severity and death outcomes (Ruszkiewicz et al., 2020). These discoveries hold great promise for our understanding variability of specific immune response to endemic SARS-CoV-2 and influenza virus infection and response to vaccination.

## VOCs Signatures Emmited by the Human Gut Microbiome and Diseases

A worldwide metagenomic study found the presence of 129 bacterial species in more than 90% of the samples from people of 195 countries (Lloyd-Price et al., 2017). The metagenome-assembled microbe genome databases have contributed to our understanding of global microbial diversity and abundance of seven main phyla—Actinobacteria, Bacteroidetes, Firmicutes, Proteobacteria, Verrucomicrobia, Fusobacteria, and Synergistetes—in the population (Lloyd-Price et al., 2017). Africa and South America people have microbiomes rich in Prevotella species and poor in Bacteroides. This Prevotella and Bacteroides antagonism correlates to population lifestyle and diet (Belizario and Napolitano, 2015; Lloyd-Price et al., 2017). Studies on differential abundance and diversity of genera and taxa in the gut microbioma have demonstrated when and how specific microbial dysbiosis—defined as loss or gain of microbiome composition or metabolic capacity—may lead to development of common human diseases (Wilkins et al., 2019). Dysbiosis either by depletion or enrichment of microbial diversity can contribute to diseases including urinary stone disease, obesity, diabetes, cardiovascular disease, and kidney disease (Wilkins et al., 2019). *Coprococcus*, *Prevotella*, and *Bacteroides* bacterial genera are predominantly enriched in the healthy populations, while the cohorts with common diseases exhibited a significant depletion of microbial genera *Bacteroides*, *Coprococcus*, *Prevotella*, *Ruminococcus*, and *Sutterella*. Hierarchal clustering revealed statistically significant similarities between diabetes and kidney diseases regarding loss of diverse protective bacterial genera (Wilkins et al., 2019).

Gut microbiota contains the most abundant microbial community, which is affected by many factors and medications such as antibiotics (Belizario and Napolitano, 2015). It was previously demonstrated that the levels of metabolites in feces, plasma, urine, and exhaled air reflect the gut homeostasis states and environmental changes in microbiome community structure (Tremaroli and Bäckhed, 2012; Wilmanski et al., 2019). The gut microbiome metabolic network is strongly altered by removal of key species or overgrowth pathogenic species (known as small intestinal bacterial overgrowth), which are associated with many gastrointestinal (GI) diseases (Clemente et al., 2012; Rowland et al., 2018). The inflammatory bowel diseases (IBD), for example, Crohn’s disease (CD) and ulcerative colitis (UC), are chronic diseases in which increases or decreases in relative abundance and diversity bacterial species can be a cause or consequence of the disease (Chang and Lin, 2016; Belizário et al., 2018). These diseases are characterized by the infiltration of neutrophils, monocytes, and lymphocytes into the intestinal lamina propria of the colon where the continued inflammatory reactions cause tissue injury, loss of goblet cells, fibrosis, erosions, and ulcerations (Chang and Lin, 2016). Studies have associated IBDs with changes in Firmicutes and Bacteroidetes ratios, and increases in Proteobacteria, Actinobacteria, in particular, within the families *Pasteurellaceae*, *Veillonellaceae*, *Fusobacteriaceae*, *Enterobacteriaceae*, and the adherent-invasive *E. coli* strains (Clemente et al., 2012; Hicks et al., 2015). Fecal samples of IBD patients had decreased *Bifidobacterium*, *Lactobacillus*, Bacteroidetes, and Actinobacteria and increased Firmicutes and Proteobacteria phyla. Clinical studies have indicated that VOCs derived from the diet and endogenous metabolism or from the microbiota metabolism can be measured through fecal and breath biopsy analyses and likely provide new option for management of these diseases (Rowland et al., 2018; Rondanelli et al., 2019).

Profiling VOCs in exhaled breath has been a strategy to finding biomarkers for GI diseases. In a study with a cohort of CD patients, 17 exhaled volatiles were identified in exhaled air that correlated with 17 bacterial taxa (Bodelier et al., 2015; Smolinska et al., 2018). In patients with active CD compared to healthy controls, the level of acetic acid in exhaled breath correlated with the abundance of *Blautia* spp. (member of Firmicutes phylum), while the increased levels of decadiene was linked to the presence of *Bacteroides* spp. (member of Bacteriodete phylum). *Clostridium citroniae* was negatively correlated to a branched alkane (C_9_C_20_) whereas other unclassified *Clostridia* spp. were related to the level of acetic acid, 1-pentanol, and n-heptane (Bodelier et al., 2015; Smolinska et al., 2018). In patients with CD in remission state, a positive correlation was observed with several Bacteriodes, including *Bacteroides uniformis* and *Prevotella copri*, and with phenol production (Smolinska et al., 2018). On the other hand, CD patients in remission state were correlated negatively with *Alistipes indistinctus*, *Bilophilia*, and *rc44* bacterial species and a reduction in the levels of methylcylclohexane (Smolinska et al., 2018). The levels of C_15_H_30_ 1-pentadecene, 3-methyl-1-butanal, octane, acetic acid, alpha-pinene, and m-cymene were elevated in active UC (Smolinska et al., 2018). Ahmed and colleagues investigated the presence of VOCs in headspace of vials containing the feces from cohorts of patients with IBS with diarrhea (IBC-D) and patients with active CD and UC (Ahmed et al., 2013). The most commonly VOCs observed in IBC-D were short chain acid cyclohexanecarboxylic acid and its derivatives, as compared to CD and UC patients. VOCs of aldehyde and ketone classes were also associated with cohort of CD patients, while 1-propanol, 2-methyl, undecane, and methoxy-phenyl-oxime were the three more abundant VOCs identified in UC cohort. An increased abundance of the following organic acids was also observed in the fecal samples of IBC-D patients: propanoic acid, butanoic acid, pentanoic acid, and hexanoic acid, as compared to healthy volunteers. The authors suggested that differential VOC production may be caused by depletion of *Lactobacilli* and *Veillonella* species and consequently dysbiosis of intestinal microbiota (Ahmed et al., 2013). It is expected that futures studies will confirm if microbiota diversity can contribute to the variation of VOCs and other biomarkers and thus may enable the monitoring and predicting of GI disease activities and their relapse.

## VOC Signatures Emitted in Bacterial Sepsis

Sepsis is characterized by dysfunction of one or multiple organs and systems in response to impartment of host immune responses to microbial infection (Haak and Wiersinga, 2017; Sjövall et al., 2017). Sepsis can be caused by various pathogens including viruses, fungi, and gram-negative or gram-positive bacteria that escaped from local site and entered into the blood, causing a systemic infection. The ultimate events in the sepsis are septic shock and multiple organ failure (Haak and Wiersinga, 2017; Sjövall et al., 2017). Neonatal and elderly populations are of greatest risk for developing sepsis. In sepsis that originated from the gut, a set of bacterial species has been frequently identified in the isolates. *S. aureus* and *S. pneumoniae* are species that predominate in gram-positive isolates, whereas *E. coli*, *Klebsiella*, and *P. aeruginosa* are common gram-negative species identified in the isolates. Different clinical criteria are used for classifying sepsis through a number of stages, including severe sepsis, septic shock, and non-septic like condition known as systemic inflammatory response syndrome (SIRS) (Haak and Wiersinga, 2017; Sjövall et al., 2017). SIRS’s most common symptoms are fever, hyperventilation, and leukocytosis. Among plasma metabolite biomarkers of SIRS, the most studied are: lactate, lactitol dehydrate, N-nonanoyl-glycine, S-phenylcysteine, and S-(3-methylbutanoyl)-dihydrolipoamide-E (Su et al., 2014; Kauppi et al., 2016). Furthermore, severity of sepsis could be determined by alterations in levels of N,N-dimethyllysine, glycerylphosphorylethanolamine, D-cysteine, and 2-phenylacetamide (Su et al., 2014; Kauppi et al., 2016). These metabolites can be end products of either host or microbial metabolism.

A central mechanism in sepsis is dysbiosis, a shift of gut microbiome composition, which can be caused by prolonged antibiotic treatment of a local infection. The investigation of the relative abundances at the phylum and class levels of the microbiome in sputum and stool samples of septic patients in ICU requires the use of next generation DNA sequencer and culture-independent techniques. On the other hand, breath biopsy has the potential to identify the bacterial richness and diversity and requires only the collection of expiratory air from patients. Examining the results presented in 51 articles, Bos and colleagues found 161 VOCs that were significantly produced during sepsis in neonates and infants (Bos et al., 2013). In the studies evaluated, Bos and colleagues discovered various microbial VOC signatures associated with one gram-positive or gram-negative species or mixed population of bacteria (Bos et al., 2013). Necrotizing enterocolitis (NEC) is a sepsis syndrome in preterm babies caused mainly by multiple species, including *Enterococcus* spp., *Staphylococcus* spp., *Sphingomonas* spp., *Escherichia* sp., and *Clostridium perfringens* (Kitsios et al., 2017; Probert et al., 2020). Fecal samples from 32 NEC from a total 1362 cases were compared with samples from frequency-matched controls without NEC. The results suggest that presence of groups of VOCs containing propanal, pentanal, and hexanol may be an earlier indicator of enterocolitis, whereas the presence of groups of VOCs containing 3-methylbutanal and 2-methylbutanal is specifically related to the leucine and isoleucine metabolism, respectively (Probert et al., 2020).

Lipopolysaccharide (LPS)—the major component of the outer membrane of gram-negative—is released from leaky gut and represents one of the primary mechanisms for induction inflammatory response and metabolic endotoxemia. Mice and rats injected with LPS purified from *E. coli* are ideal models for the study of inflammation and systemic sepsis (Bos et al., 2013; Langeroudi et al., 2014). In one study with septic mice induced by LPS, it was observed that there were increased levels of carbon monoxide (CO) and ratio of CO to CO_2_ in a dose-responsive manner within hours after injection (Langeroudi et al., 2014). A study with 18 healthy volunteers who received 2 ng *E. coli* LPS kg^-1^ body weight intravenously demonstrated that all volunteers developed SIRS like symptoms (Peters et al., 2017). The exhaled VOC concentrations of 3-methyl-pentane, 4-methyl-pentanol, 1-hexanol, 2,4-dimethyl-heptane, decane, and one unknown compound changed significantly after LPS infusion. Only the unknown compound was directly associated with variations of plasma levels of IL-6, which is a biomarker for inflammation. A report by Fink and co-workers examined the variation in the levels of VOCs in exhaled breath in different rat model of sepsis induced by cecal ligation, LPS administration, an inflammatory stimulus, and hemorrhagic shock induced by rapid arterial blood withdrawal (Fink et al., 2015). In this study, direct detection of VOCs was done by multicapillary column ion-mobility spectrometry (MCC-IMS). The levels of acetone reduced in all rodent sepsis models. Endotoxemic and septic rats compared with sham rats had significant differences in the release of butanal, 3-pentanone, and 2-hexanone. All these VOCs declined in the course of experiment. The authors suggested that differential changes in plasma metabolites and VOCs in exhaled breath may be caused by gut-origin infection and other pulmonary processes. The results partially confirm the findings observed in humans undergoing inflammatory, sepsis, and septic shock processes.

## Conclusion and Remarks

The discovery of the complex interface between the host and its own personalized microbioma (bacteria, virus, parasites, yeasts) has changed the way we evaluate healthy and diseased humans. Microbiota display different metabolic pathways to provide critical nutritional support to organs and tissues of human body. Dysbiosis after a microbial infection leads to installation of host inflammatory response and production of multiple chemical signals from host and microbes. Recent studies have confirmed that specific VOC microbial signatures may help to diagnose and monitor the bacterial and virus infection as well as to monitor the host response to biological and chemo therapeutics. In future ion-mobility spectroscopy or proton–ion reaction mass spectrometry approaches will allow online real‐time detection and quantification of VOCs for target and non-target analyses in routine and large clinical studies correlating healthy and diseased states. Exploring commensal and pathogenic bacteria species interaction *via* their chemical products (metabolites) is crucial to elucidate their biological significance and mechanisms behind the connected network between microbes–microbes and microbes–host cells.

## Author Contributions

JB, JF, and MM conducted the literature review process and selected articles by grading, and categorizing criteria, and quality of articles. JB and MM wrote the text and prepared figures and table, and JF edited and revised the article. All authors contributed to the article and approved the submitted version.

## Funding

The authors are supported by grants from Fundação de Amparo a Pesquisa do Estado de São Paulo (FAPESP, proc. 2015/1177-8, 2015/18647-6, 2018/24922-8, 2007/04513-1 2018/22960-0) and Conselho Nacional de Desenvolvimento Científico e Tecnológico (CNPq proc 486048/2011 and 312206/2016-0).

## Conflict of Interest

The authors declare that the research was conducted in the absence of any commercial or financial relationships that could be construed as a potential conflict of interest.

## References

[B1] Abd El Qader A. Lieberman D. Shemer Avni Y. Svobodin N. Lazarovitch T. Sagi O. (2015). Volatile organic compounds generated by cultures of bacteria and viruses associated with respiratory infections. Biomed. Chromatogr. 29 (12), 1783–1790. 10.1002/bmc.3494 26033043

[B2] Abdullah A. A. Altaf-Ul-Amin M. Ono N. Sato T. Sugiura T. Morita A. H. (2015). Development and mining of a volatile organic compound database. Biomed. Res. Int. 2015, 139254. 10.1155/2015/139254 26495281 PMC4606137

[B3] Ahmed I. Greenwood R. Costello B. D. L. Ratcliffe N. M. Probert C. S. (2013). An investigation of fecal volatile organic metabolites in irritable bowel syndrome. PLoS One 8 (3), e58204. 10.1371/journal.pone.0058204 23516449 PMC3596408

[B4] Ahmed W. Lawal O. Nijsen T. M. Goodacre R. Fowler S. J. (2017). Exhaled volatile organic compounds of infection: a systematic review. ACS Infect. Dis. 3 (10), 695–710. 10.1021/acsinfecdis.7b00088 28870074

[B5] Ahmed W. M. Brinkman P. Weda H. Knobel H. H. Xu Y. Nijsen T. M. (2018). Methodological considerations for large-scale breath analysis studies: lessons from the U-BIOPRED severe asthma project. J. Breath Res. 13 (1), 016001. 10.1088/1752-7163/aae557 30272570

[B6] Al-Saiedy M. Gunasekara L. Green F. Pratt R. Chiu A. Yang A. (2018). Surfactant dysfunction in ARDS and bronchiolitis is repaired with cyclodextrins. Mil. Med. 183 (suppl_1), 207–215. 10.1093/milmed/usx204 29635617 PMC6544870

[B7] Amann A. Costello B de L. Miekisch W. Schubert J. Buszewski B. Pleil J. (2014). The human volatilome: volatile organic compounds (VOCs) in exhaled breath, skin emanations, urine, feces and saliva. J. Breath Res. 8 (3), 34001. 10.1088/1752-7155/8/3/034001 24946087

[B8] Arasaradnam R. P. Ouaret N. Thomas M. G. Quraishi N. Heatherington E. Nwokolo C. U. (2013). A novel tool for noninvasive diagnosis andtracking of patients with inflammatory bowel disease. Inflamm. BowelDis. 19, 999–1003. 10.1097/MIB.0b013e3182802b26 23478806

[B9] Audrain B. Farag M. A. Ryu C.-M. Ghigo J.-M. (2015). Role of bacterial volatile compounds in bacterial biology. FEMS Microbiol. Rev. 39 (2), 222–233. 10.1093/femsre/fuu013 25725014

[B10] Barnes P. J. (2017). Cellular and molecular mechanisms of asthma and COPD. Clin. Sci. (Lond) 131, 1541–1558. 10.1042/CS20160487 28659395

[B11] Belizario J. E. Napolitano M. (2015). Microbiomes and their roles in dysbiosis, common diseases and novel therapeutic approaches. Front. Microbiol. 6, 1050. 10.3389/fmicb.2015.01050 26500616 PMC4594012

[B12] Belizário J. E. Faintuch J. Garay-Malpartida M. (2018). Gut microbiome dysbiosis and immunometabolism: new frontiers for treatment of metabolic diseases. Mediators Inflamm. 2018, 2037838. 10.1155/2018/2037838 30622429 PMC6304917

[B13] Belizario J. E. Sulca-Lopez M. Sircili M. Faintuch J. (2020). “Role of small volatile signaling molecules in the regulation of bacterial antibiotic resistance and quorum sensing systems,” in Trends in Quorum Sensing and Quorum Quenching: New Perspectives and Applications. Eds. Rai R. Bai J. (Boca Raton, FL, USA: CRC Press, Taylor & Francis), pp. 215–pp. 223. 10.1201/9780429274817

[B14] Bodelier A. G. Smolinska A. Baranska A. Dallinga J. W. Mujagic Z. Vanhees K. (2015). Volatile organic compounds in exhaled air as novel marker for disease activity in Crohn’s disease: a metabolomic approach Inflamm. Bowel Dis. 21 (8), 1776–1785. 10.1097/MIB.0000000000000436 26199990

[B15] Boots A. W. van Berkel J. J. Dallinga J. W. Smolinska A. Wouters E. F. van Schooten F. J. (2012). The versatile use of exhaled volatile organic compounds in human health and disease. J. Breath Res. 6, 27108. 10.1088/1752-7155/6/2/027108 22621865

[B16] Bos L. D. Weda H. Wang Y. Knobel H. H. Nijsen T. M. Vink T. J. (2014). Exhaled breath metabolomics as a noninvasive diagnostic tool for acute respiratory distress syndrome. Eur. Respir. J. 44 (1), 188–197. 10.1183/09031936.00005614 24743964

[B17] Bos L. D. Sterk P. J. Fowler S. J. (2016). Breathomics in the setting of asthma and chronic obstructive pulmonary disease. J. Allergy Clin. Immunol. 138 (4), 970–976. 10.1016/j.jaci.2016.08.004 27590400

[B18] Bos L. D. van Walree I. C. Kolk A. H. Janssen H. G. Sterk P. J. Schultz M. J. (2013). Alterations in exhaled breath metabolite mixtures in two rat models of lipopolysaccharide-induced lung injury. J. Appl. Physiol. 115, 1487–1495. 10.1152/japplphysiol.00685.2013 23908314

[B19] Bos L. D. J. Sterk P. J. Schultz M. J. (2013). Volatile metabolites of pathogens: a systematicreview. PLoS Pathog. 9 (5), e1003311. 10.1371/journal.ppat.1003311 23675295 PMC3649982

[B20] Brinkman P. van de Pol M. A. Gerritsen M. G. Bos L. D. Dekker T. Smids B. S. (2015). Exhaled breath profiles in the monitoring of loss of control and clinical recovery in asthma. Clin. Exp. Allergy 47 (9), 1159–1169. 10.1111/cea.12965 28626990

[B21] Brinkman P. Zee A. M. Wagener A. H. (2019). Breathomics and treatable traits for chronic airway diseases. Curr. Opin. Pulm. Med. 25 (1), 94–100. 10.1097/MCP.0000000000000534 30325789

[B22] Broza Y. Y. Zuri L. Haick H. (2014). Combined volatolomics for monitoring of human body chemistry. Sci. Rep. 4, 4611. 10.1038/srep04611 24714440 PMC3980217

[B23] Chang C. Lin H. (2016). Dysbiosis in gastrointestinal disorders. Best Pract. Res. Clin. Gastroenterol. 30 (1), 3–15. 10.1016/j.bpg.2016.02.001 27048892

[B24] Clemente J. C. Ursell L. K. Parfrey L. W. Knight R. (2012). The impact of the gut microbiota on human health: an integrative view. Cell 148 (6), 1258–1270. 10.1016/j.cell.2012.01.035 22424233 PMC5050011

[B25] de Lacy Costello B. Amann A. Al-Kateb H. Flynn C. Filipiak W. Khalid T. (2014). A review of the volatiles from the healthy human body. J. Breath Res. 8 (1), 14001. 10.1088/1752-7155/8/1/014001 24421258

[B26] Doran S. L. F. Romano A. Hanna G. B. (2018). Optimisation of sampling parameters for standardised exhaled breath sampling. Breath Res. 12 (2018), 016007. 10.1088/1752-7163/aa8a46 29211685

[B27] Dragonieri S. Pennazza G. Carratu P. Resta O. (2017). Electronic nose technology in respiratory diseases. Lung 195, 157–165. 10.1007/s00408-017-9987-3 28238110

[B28] Filipiak W. Sponring A. Bauer M. Filipiak A. Ager C. Wiesenhofer H. (2012). Molecular analysis of volatile metabolites released specifically by *Staphylococcus aureus* and *Pseudomonas aeruginosa* . BMC Microbiol. 12, 113. 10.1186/1471-2180-12-113 22716902 PMC3444334

[B29] Fink T. Wolf A. Maurer F. Albrecht F. W. Heim N. Wolf B. (2015). Volatile organic compounds during inflammation and sepsis in rats: a potential breath test using ion-mobility spectrometry. Anesthesiology 122 (1), 117–126. 10.1097/ALN.0000000000000420 25170570

[B30] Gilchrist F. J. Belcher J. Jones A. M. Smith D. Smyth A. R. Southern K. W. (2015). Exhaled breath hydrogen cyanide as a marker of early *Pseudomonas aeruginosa* infection in children with cystic fibrosis. ERJ Open Res. 1 (2), 00044–02015. 10.1183/23120541.00044-2015 27730156 PMC5005121

[B31] Gowda H. Ivanisevic J. Johnson C. H. Kurczy M. E. Benton H. P. Rinehart D. (2014). Interactive XCMS Online: simplifying advanced metabolomic data processing and subsequent statistical analyses. Anal. Chem. 86 (14), 6931–6939. 10.1021/ac500734c 24934772 PMC4215863

[B32] Haak B. W. Wiersinga W. J. (2017). The role of the gut microbiota in sepsis. Lancet Gastroenterol. Hepatol. 2 (2), 135–143. 10.1016/S2468-1253(16)30119-4 28403983

[B33] Herbig J. Beauchamp J. (2014). Towards standardization in the analysis of breath gas volatiles. J. Breath Res. 8 (2014), 037101. 10.1088/1752-7155/8/3/037101 25189420

[B34] Hicks L. C. Huang J. Kumar S. Powles S. T. Orchard T. R. Hanna G. H. (2015). Analysis of exhaled breath volatile organic compounds in inflammatory bowel disease: a pilot study. J. Crohns Colitis 9 (9), 731–737. 10.1093/ecco-jcc/jjv102 26071410

[B35] Huffnagle G. B. Dickson R. P. Lukacs N. W. (2017). The respiratory tract microbiome and lung inflammation: a two-way street. Mucosal Immunol. 10 (2), 299–306. 10.1038/mi.2016.108 27966551 PMC5765541

[B36] Hüppe T. Lorenz D. Wachowiak M. Maurer F. Meiser A. Groesdonk H. (2017). Volatile organic compounds in ventilated critical care patients: a systematic evaluation of cofactors. BMC Pulm. Med. 17 (1), 116. 10.1186/s12890-017-0460-0 28830533 PMC5567647

[B37] Ibrahim B. Basanta M. Cadden P. Singh D. Douce D. Woodcock A. (2011). Noninvasive phenotyping using exhaled volatile organic compounds in asthma. Thorax 66, 804–809. 10.1136/thx.2010.156695 21749985

[B38] Kauppi A. M. Edin A. Ziegler I. Mölling P. Sjöstedt A. Gylfe Å. (2016). Metabolites in blood for prediction of bacteremic sepsis in the emergency room. PLoS One 11 (1), e0147670. 10.1371/journal.pone.0147670 26800189 PMC4723089

[B39] Kitsios G. D. Morowitz M. J. Dickson R. P. Huffnagle G. B. McVerry B. J. Morris A. (2017). Dysbiosis in the intensive care unit: microbiome science coming to the bedside. J. Crit. Care 38, 84–91. 10.1016/j.jcrc.2016.09.029 27866110 PMC5328797

[B40] Küntzel A. Oertel P. Fischer S. Bergmann A. Trefz P. Schubert J. (2018). Comparative analysis of volatile organic compounds for the classification and identification of mycobacterial species. PLoS One 13 (3), e0194348. 10.1371/journal.pone.0194348 29558492 PMC5860768

[B41] Kuruvilla M. E. Lee F. E. Lee G. B. (2019). Understanding asthma phenotypes, endotypes, and mechanisms of disease. Clin. Rev. Allergy Immunol. 56 (2), 219–233. 10.1007/s12016-018-8712-1 30206782 PMC6411459

[B42] Lamarche D. Johnstone J. Zytaruk N. Clarke F. Hand L. Loukov D. (2018). Microbial dysbiosis and mortality during mechanical ventilation: a prospective observational study. Respir. Res. 19 (1), 245. 10.1186/s12931-018-0950-5 30526610 PMC6286574

[B43] Langeroudi A. G. Hirsch C. M. Estabragh A. S. Meinardi S. Blake D. R. Barbour A. G. (2014). Elevated carbon monoxide to carbon dioxide ratio in the exhaled breath of mice treated with a single dose of lipopolysaccharide. Open Forum Infect. Dis. 1 (2), 1–8. 10.1093/ofid/ofu085 PMC4281777 25734151

[B44] Lee J. Jayaraman A. Wood T. K. (2007). Indole is an inter-species biofilm signal mediated by SdiA. BMC Microbiol. 7, 42. 10.1186/1471-2180-7-42 17511876 PMC1899176

[B45] Lemfack M. C. Gohlke B. O. Toguem S. M. T. Preissner S. Piechulla B. Preissner R. (2017). mVOC 2.0: a database of microbial volatiles. Nucleic Acids Res. 46 (D1), D1261–D1265. 10.1093/nar/gkx1016 PMC5753297 29106611

[B46] Leopold J. H. Philipp A. Bein T. Redel A. Gruber M. Schultz M. J. (2019). Volatile organic compound profiles in outlet air from extracorporeal life-support devices differ from breath profiles in critically ill patients. ERJ Open Res. 5 (2), 00134–2018. 10.1183/23120541.00134-2018 PMC6441674 30949490

[B47] Lim S. H. Martino R. Anikst V. Xu Z. Mix S. Benjamin R. (2016). Rapid Diagnosis of Tuberculosis from Analysis of Urine Volatile Organic Compounds. ACS Sens. 1 (7), 852–856. 10.1021/acssensors.6b00309 29057329 PMC5648341

[B48] Lloyd-Price J. Mahurkar A. Rahnavard G. Crabtree J. Orvis J. Hall A. B. (2017). Strains, functions and dynamics in the expanded Human Microbiome Project. Nature 550 (7674), 61–66. 10.1038/nature23889 28953883 PMC5831082

[B49] Mashir A. Paschke K. M. van Duin D. Shrestha N. K. Laskowski D. Storer M. K. (2011). Effect of the influenza A (H1N1) live attenuated intranasal vaccine on nitric oxide (FE(NO)) and other volatiles in exhaled breath. J. Breath Res. 5 (3):37107. 10.1088/1752-7155/5/3/037107 PMC4552053 21757798

[B50] Mendez R. Banerjee S. Bhattacharya S. K. Banerjee S. (2019). Lung inflammation and disease: A perspective on microbial homeostasis and metabolism. IUBMB Life. 71 (2), 152–165. 10.1002/iub.1969 30466159 PMC6352907

[B51] Moffatt M. F. Cookson W. O. (2017). The lung microbiome in health and disease. Clin. Med. (Lond.) 17 (6), 525–529. 10.7861/clinmedicine.17-6-525 29196353 PMC6297685

[B52] Molyneaux P. L. Mallia P. Cox M. J. Footitt J. Willis-Owen S. A. Homola D. (2013). Outgrowth of the bacterial airway microbiome after rhinovirus exacerbation of chronic obstructive pulmonary disease. Am. J. Respir. Crit. Care Med. 188 (10), 1224–1231. 10.1164/rccm.201302-0341OC 23992479 PMC3863728

[B53] Neerincx A. H. Vijverberg S. J. H. Bos L. D. J. Brinkman P. van der Schee M. P. de Vries R. (2017). Breathomics from exhaled volatile organic compounds in pediatric asthma. Pediatr. Pulmonol. 52 (12), 1616–1627. 10.1002/ppul.23785 29082668

[B54] Nizio K. D. Perrault K. A. Troobnikoff A. N. Ueland M. Shoma S. Iredell J. R. (2016). In vitro volatile organic compound profiling using GC×GC-TOFMS to differentiate bacteria associated with lung infections: a proof-of-concept study. J. Breath Res. 10 (2), 26008. 10.1088/1752-7155/10/2/026008 27120170

[B55] O’Connor E. M. (2013). The role of gut microbiota in nutritional status. Curr. Opin. Clin. Nutr. Metab. Care 16 (5), 509–516. 10.1097/MCO.0b013e3283638eb3 23852088

[B56] Palma S. I. C. J. Traguedo A. P. Porteira A. R. Frias M. J. Gamboa H. Roque A. C. A. (2018). Machine learning for the meta analyses of microbial pathogens’ volatile signatures. Sci. Rep. 8, 3360. 10.1038/s41598-018-21544-1 29463885 PMC5820279

[B57] Peters A. L. Gerritsen M. G. Brinkman P. Zwinderman K. A. H. Vlaar A. P. J. Bos L. D. (2017). Volatile organic compounds in exhaled breath are independent of systemic inflammatory syndrome caused by intravenous lipopolysaccharide infusion in humans: results from an experiment in healthy volunteers. J. Breath Res. 11 (2), 026003. 10.1088/1752-7163/aa6545 28397711

[B58] Phillips M. Cataneo R. N. Greenberg J. Grodman R. Gunawardena R. Naidu A. (2003). Effect of oxygen on breath markers of oxidative stress. Eur. Respir. J. 21, 48–51. 10.1183/09031936.02.00053402 12570108

[B59] Phillips M. Cataneo R. N. Chaturvedi A. Danaher P. J. Devadiga A. Legendre D. (2010). Effect of influenza vaccination on oxidative stress products in breath. J. Breath Res. 4 (2), 26001. 10.1088/1752-7155/4/2/026001 21383469

[B60] Phillips M. Cataneo R. N. Chaturvedi A. Kaplan P. D. Libardoni M. Mundada M. (2013). Detection of an extended human volatome with comprehensive two-dimensional gas chromatography time-of-flight mass spectrometry. PLoS One 8 (9), e75274. 10.1371/journal.pone.0075274 24086492 PMC3783494

[B61] Probert C. Greenwood R. Mayor A. Hughes D. Aggio R. Jackson R. E. (2020). Faecal volatile organic compounds in pretermbabies at risk of necrotising enterocolitis: the DOVE study. Arch. Dis.Child Fetal Neonatal 105 (5), 474–479. 10.1136/archdischild-2019-318221. Ed. fetalneonatal-2019-318221. 31871055

[B62] Ratiu I. A. Ligor T. Bocos-Bintintan V. Buszewski B. (2017). Mass spectrometric techniques for the analysis of volatile organic compounds emitted from bacteria. Bioanalysis 9 (14), 1069–1092. 10.4155/bio-2017-0051 28737423

[B63] Rees C. A. Burklund A. Stefanuto P. H. Schwartzman J. D. Hill J. E. (2018). Comprehensive volatile metabolic fingerprinting of bacterial and fungal pathogen groups. J. Breath Res. 12 (2), 026001. 10.1088/1752-7163/aa8f7f 28952968 PMC5832594

[B64] Ribet D. Cossart P. (2015). How bacterial pathogens colonize their hosts and invade deeper tissues. Microbes Infect. 17 (3), 173–183. 10.1016/j.micinf.2015.01.004 25637951

[B65] Rolla G. Guida G. Heffler E. (2007). Diagnostic classification of persistent rhinitisand its relationship to exhaled nitric oxide and asthma: a clinical study of a consecutive series of patients. Chest 131 (5), 1345–1352. 10.1378/chest.06-2618 17317733

[B66] Rondanelli M. Perdoni F. Infantino V. Faliv M. A. Peroni G. Iannello G. (2019). Volatile organic compounds as biomarkers of gastrointestinal diseases and nutritional status. J. Anal. Methods Chem. 2019, 7247802. 10.1155/2019/7247802 31583160 PMC6754926

[B67] Rosas-Salazar C. Shilts M. H. Tovchigrechko A. Schobel S. Chappell J. D. Larkin E. K. (2016). Differences in the nasopharyngeal microbiomeduring acute respiratory tract infection with human rhinovirus and respiratory syncytial virus ininfancy. J. Infect. Dis. 214, 1924–1928. 10.1016/j.jaci.2017.10.049 27923952 PMC5142087

[B68] Rowland I. Gibson G. Heinken A. Scott K. Swann J. Thiele I. (2018). Gut microbiota functions: metabolism of nutrients and other food components. Eur. J. Nutr. 57 (1), 1–24. 10.1007/s00394-017-1445-8 PMC5847071 28393285

[B69] Ruszkiewicz D. M. Sanders D. O’Brien R. Hempel F. Reed M. J. Riepe A. C. (2020). Diagnosis of COVID 19 by analysis of breathwith gas chromatography ion mobility spectrometry: a feasibility study. EClinicalMedicine 24, 100609. 10.1016/j.eclinm.2020.100609 PMC7585499 33134902

[B70] Rutherford S. T. Bassler B. L. (2012). Bacterial quorum sensing: its role in virulence and possibilities for its control. Cold Spring Harb. Perspect. Med. 2 (11), a012427. 10.1101/cshperspect.a012427 23125205 PMC3543102

[B71] Serrano A. G. Pérez-Gil J. (2006). Protein-lipid interactions and surface activity in the pulmonary surfactant system. Chem. Phys. Lipids 141 (1-2), 105–118. 10.1016/j.chemphyslip.2006.02.017 16600200

[B72] Sethi S. Nanda R. Chakraborty T. (2013). Clinical application of volatile organic compound analysis for detecting infectious diseases. Clin. Microbiol. Rev. 26, 462–476. 10.1128/CMR.00020-13 23824368 PMC3719490

[B73] Shatalin K. Shatalina E. Mironov A. Nudler E. (2011). H2S: a universal defense against antibiotics in bacteria. Science 334 (6058), 986–990. 10.1126/science.1209855 22096201

[B74] Sjövall F. Perner A. Hylander Møller M. (2017). Empirical mono versus combination antibiotic therapy in adult intensive care patients with severe sepsis – A systematic review with meta-analysis and trial sequential analysis. J. Infect. 74, 331–344. 10.1016/j.jinf.2016.11.013 27919645

[B75] Smolinska A. Hauschild A. C. Fijten R. R. Dallinga J. W. Baumbach J. van Schooten F. J. (2014). Current breathomics - a review on data pre-processing techniques and machine learning in metabolomics breath analysis. J. Breath Res. 8 (2), 27105. 10.1088/1752-7155/8/2/027105 24713999

[B76] Smolinska A. Tedjo D. I. Blanchet L. Bodelier A. Pierik M. J. Masclee A. A. M. (2018). Volatile metabolites in breath strongly correlate with gut microbiome in CD patients. Anal. Chim. Acta 1025, 1–11. 10.1016/j.aca.2018.03.046 29801597

[B77] Stavropoulos G. Jonkers D. M. A. E. Mujagic Z. Mujagic Z. Koek G. D. Masclee A. D. M. (2020). Implementation of quality controls is essential to prevent batch effects in breathomics data and allow for cross-study comparisons. J. Breath Res. 14 (2), 026012. 10.1088/1752-7163/ab7b8d 32120348

[B78] Su L. Huang Y. Zhu Y. Xia L. Wang R. Xiao K. (2014). Discrimination of sepsis stage metabolic profiles with an LC/MS-MS based metabolomics approach. BMJ Open Respir. Res. 1 (1), e000056. 10.1136/bmjresp-2014-000056. 10. PMC4265126 25553245

[B79] Timm C. M. Lloyd E. P. Egan A. Mariner R. Karig D. (2018). Direct growth of bacteria in headspace vials allows for screening of volatiles by gas chromatography mass spectrometry. Front. Microbiol. 9, 491. 10.3389/fmicb.2018.00491 29662472 PMC5890184

[B80] Tremaroli V. Bäckhed F. (2012). Functional interactions between the gut microbiota and host metabolism. Nature 489, 242–249. 10.1038/nature11552 22972297

[B81] Ulanowska A. Kowalkowski T. Hrynkiewicz K. Jackowski M. Buszewski B. (2011). Determination of volatile organic compounds in human breath for Helicobacter pylori detection by SPMEGC/MS. Biomed. Chromatogr. 25 (3), 391–397. 10.1002/bmc.1460 21321973

[B82] van Oort P. M. P. Nijsen T. Weda H. Knobel H. Dark P. Felton T. (2017). BreathDx – molecular analysis of exhaled breath as a diagnostic test for ventilator–associated pneumonia: protocol for a European multicentre observational study. BMC Pulm. Med. 17 (1), 1. 10.1186/s12890-016-0353-7 28049457 PMC5210294

[B83] van Vliet D. Smolinska A. Jöbsis Q. Rosias P. Muris J. Dallinga J. (2017). Can exhaled volatile organic compounds predict asthma exacerbations in children? J. Breath Res. 11 (1), 016016. 10.1088/1752-7163/aa5a8b 28102830

[B84] Vinaixa M. Schymanski E. L. Neumann S. Navarro M. Salek R. M. Yanes O. (2016). Mass spectral databases for LC/MS and GC/MS-based metabolomics: State of the field and future prospects. Trends Analyt. Chem. 78, 23–35. 10.1007/978-1-4939-7819-9_14

[B85] Vizcaíno J. A. Csordas A. del-Toro N. Dianes J. A. Griss J. Lavidas I. (2016). 2016 update of the PRIDE database and its related tools. Nucleic Acids Res. 44 (D1), D447–D456. 10.1093/nar/gkv1145 26527722 PMC4702828

[B86] Walter J. M. Wilson J. Ware L. B. (2014). Biomarkers in acute respiratory distress syndrome: from pathobiology to improving patient care. Expert Rev. Respir. Med. 8 (5), 573–586. 10.1586/17476348.2014.924073 24875533

[B87] Wilkins L. J. Monga M. Miller A. W. (2019). Defining dysbiosis for a cluster of chronic diseases. Sci. Rep. 9 (1), 12918. 10.1038/s41598-019-49452-y 31501492 PMC6733864

[B88] Wilmanski T. Rappaport N. Earls J. C. Magis A. T. Manor O. Lovejoy J. (2019). Blood metabolome predicts gut microbiome α-diversity in humans. Nat. Biotechnol. 37 (10), 1217–1228. 10.1038/s41587-019-0233-9 31477923

[B89] Wilson A. D. (2015). Advances in electronic-nose technologies for the detection of volatile biomarker metabolites in the human breath. Metabolites 5 (1), 140–163. 10.3390/metabo5010140 25738426 PMC4381294

